# Acute Respiratory Failure Secondary to Low-Dose Opioid Administration in a Patient With Obstructive Sleep Apnea and Obesity Hypoventilation Syndrome After Undergoing Trans-sphenoidal Tumor Resection

**DOI:** 10.7759/cureus.56973

**Published:** 2024-03-26

**Authors:** Dennys Rivera, Adrian B Muniz-Sarriera, Joshua Marcial, Hector Torres, Elfren Colón-Rodríguez, Maria J Crespo

**Affiliations:** 1 Anesthesiology, University of Puerto Rico, Medical Sciences Campus, San Juan, USA; 2 Physiology and Anesthesiology, University of Puerto Rico, Medical Sciences Campus, San Juan, USA

**Keywords:** opioids, respiratory failure, multimodal analgesia, obesity hypoventilation syndrome (ohs), obstructive sleep apnea (osa)

## Abstract

We present a case of an obese 56-year-old male with obstructive sleep apnea (OSA), obesity hypoventilation syndrome (OHS), and pituitary macroadenoma, who underwent nasal endoscopic trans-sphenoidal resection. Surgery was performed under general anesthesia, uneventfully as planned. The patient experienced, however, delayed emergence despite receiving adequate neuromuscular blockade agent reversal. Extubation was performed and the patient was transferred to the recovery room on a Venturi mask (50% fraction of inspired oxygen, FIO2)and 93% saturation. Postoperatively, the patient was complaining of acute pain that did not resolve with non-opioid medications and a low morphine dose (0.035 mg/kg) for pain management was administered. Subsequently, he developed severe respiratory depression, requiring intubation. After three hours, the patient was extubated, transferred to the intensive care unit, and discharged five days later. Although the patient recovered favorably, this case highlights the risks of administering opioids to patients with OSA and OHS. To our knowledge, this is the first case reporting extreme respiratory depression secondary to the administration of a very low dose of morphine in patients with these comorbidities. Therefore, it is essential to be cautious with the use of opioids and to explore multimodal pain relief methods for these patients.

## Introduction

Obstructive sleep apnea (OSA) is a disorder that causes frequent interruptions in breathing during sleep due to the obstruction of the upper airway. These disturbances can cause decreased oxygen levels and disrupted sleep, leading to daytime fatigue and drowsiness [1]. OSA severity can be assessed by the apnea-hypopnea index (AHI), which represents the average number of apneas and hypopneas per hour of sleep. When the AHI is equal to, or greater than, 5, the patient is diagnosed with OSA [2]. In the United States, approximately 25-33% of men and around 9-17% of women fulfill the criteria for OSA [3]. Individuals suspected of having OSA are approximately 2.5 times more likely to develop postoperative respiratory failure, and more than 1.5 times more likely to have a cardiac event than individuals without OSA symptoms [4].

Obesity hypoventilation syndrome (OHS) is defined as a combination of obesity (BMI > 30 kg/m²) and high carbon dioxide levels (PaCO_2_ > 45 mmHg) during the day, in the absence of other underlying reasons for reduced ventilation [5]. Approximately 90% of individuals diagnosed with OHS also suffer from OSA [6]. Although OHS has been linked to increased excess morbidity and mortality, it is not standard practice to screen high-risk individuals for this condition before surgery [7]. There is a significant lack of comprehensive information in the anesthesiology literature on the perioperative assessment and management of patients with OSA and OHS. As the global obesity epidemic continues, and considering that the incidence of OHS is likely to increase, it is imperative for anesthesiologists to identify and effectively manage patients afflicted with this syndrome [8].

Surgery carries a substantial risk of respiratory failure in morbidly obese patients with hypercapnia due to OSA and OHS [1]. At present, there are no established guidelines or consensus for managing OSA and OHS patients undergoing transsphenoidal surgery. These patients face the potential risk of opioid-induced ventilatory impairment (OIVI) [1].

We present a 56-year-old male with obesity, OSA, OHS, and pituitary macroadenoma who underwent a trans-sphenoidal tumor resection surgery. In postoperative care, he experienced severe respiratory depression requiring intubation secondary to the administration of a low dose of morphine. This case underscores the importance of opioid-sparing strategies in patients with OSA and OHS.

## Case presentation

The patient was a 56-year-old male with American Society of Anesthesiology (ASA) status 3, who had OSA, obesity class II (BMI 39.5 kg/m^2^), and hypertension. The cardiac physical examination was unremarkable. Although a preoperative echocardiogram is indicated in patients with concomitant OSA and OHS, this procedure was not performed because the patient was diagnosed with OHS postoperatively. He presented with peripheral vision loss in his left eye, and nasal obstruction, predominantly on the right side, accompanied by facial pressure and hyposmia. The patient's follow-up included magnetic resonance imaging (MRI) of the head, which revealed a pituitary non-functional adenoma. Therefore, the patient underwent a nasal endoscopic trans-sphenoidal macroadenoma resection performed by neurosurgery and otorhinolaryngology (ENT) services.

The patient was positioned supinely, monitored according to ASA standards during the intraoperative period, and premedicated with midazolam (0.10 mg/kg). The general anesthesia induction was performed with propofol (2.5 mg/kg) and maintained with sevoflurane. Fentanyl (300 mcg IV) was also administered intraoperatively in refracted doses that were calculated using lean body weight (LBW). Body temperature and end tidal CO_2_ (ETCO_2_) were maintained at 36 °C and 30-35 mmHg, respectively. Surgery, which lasted six hours, was completed without any complications. The patient experienced a delayed emergence during the postoperative period; however, despite receiving adequate paralytic reversal with sugammadex (4 mg/kg). Flumazenil (0.2 mg) and naloxone (0.04 mg) were also administered to reverse the effects of midazolam and fentanyl, respectively. Initially, these drugs did not produce the desired response, and additional doses were administered, while airway assistance was provided until the patient emerged completely from the anesthesia. Subsequently, the patient was extubated and transferred to the post-anesthesia care unit (PACU) where he received oxygen therapy through a venturi mask set to deliver a 50% oxygen concentration. The patient's oxygen saturation level (SpO_2_) was 93%. At this moment, white blood cells, red blood cells, and hemoglobin values were 11.91 × 103/µL, 4.33 × 106/µL, and 12.2 g/dl, respectively.

**Figure 1:**
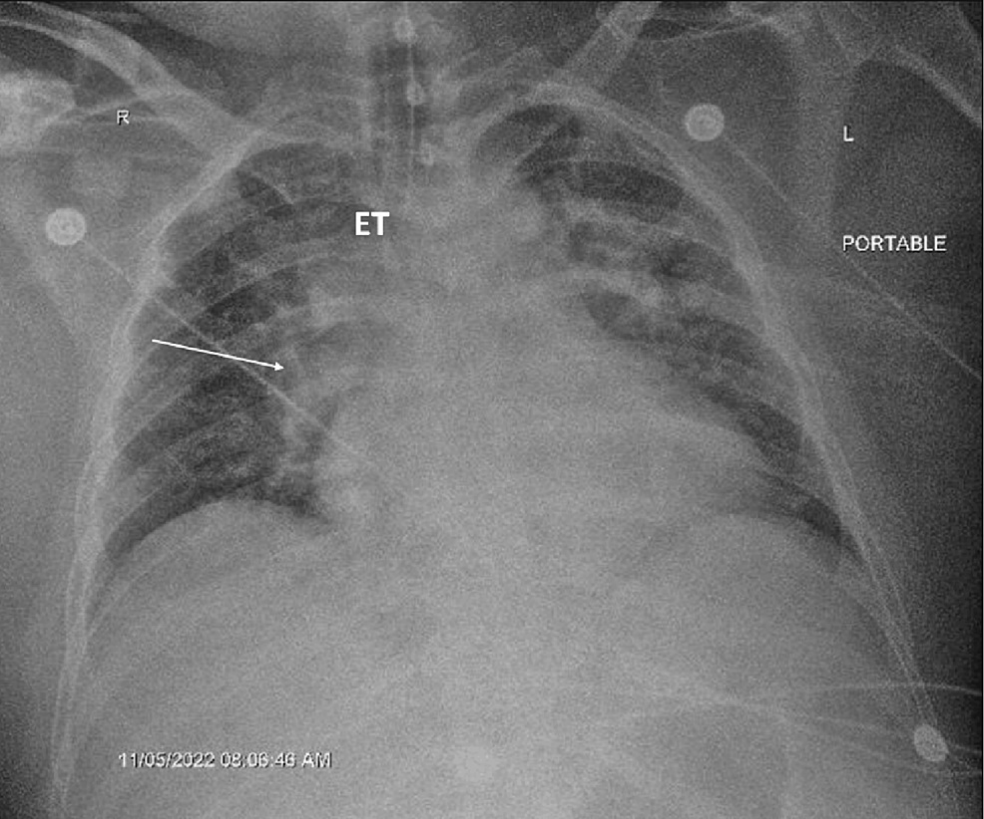
Chest X-ray showing slight engorgement of the pulmonary vasculature (arrow) and the endotracheal tube (ET) tip located in the lower trachea after the patient’s intubation in the post-anesthesia care unit

**Figure 2:**
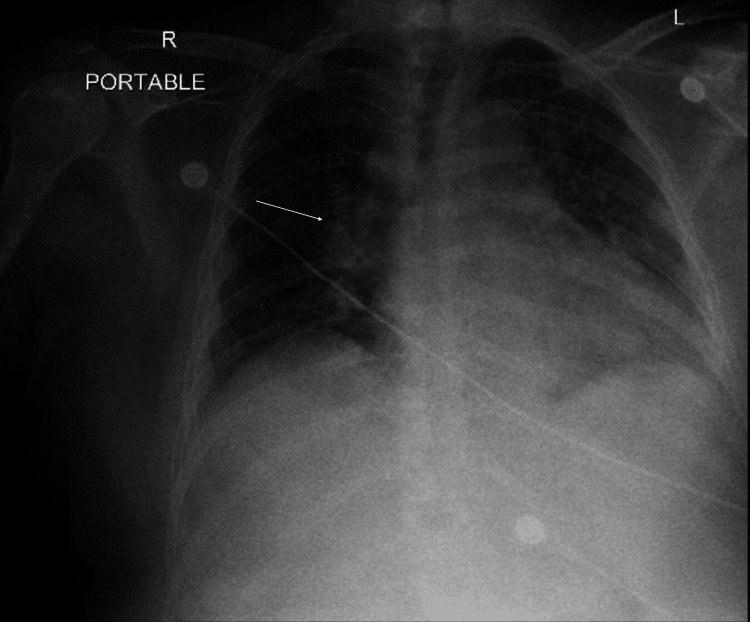
Chest X-ray showing improvement in the status of the pulmonary vasculature (arrow) and clear lung fields three days after the patient’s intubation in the post-anesthesia care unit

Six hours after the surgery, the patient was alert, with the venturi mask maintain a SpO_2_ level above 95%. Despite receiving oral acetaminophen (1000 mg) and intravenous ketorolac (15 mg) for pain management, the patient continued to experience acute pain. Thus, a low dose of morphine (0.035 mg/kg) was administered. Following this administration, he became diaphoretic and somnolent. The SpO_2_ dropped to 86%, at which time he began to hypo-ventilate. An additional dose of naloxone (0.04 mg) was then administered, which improved his respiratory status. One hour later, however, the patient not only became somnolent and diaphoretic again, but his oxygen saturation dropped to 78%, his blood pressure rose to 220/98 mm Hg, and his heart rate rose to 97 bpm. At this point, the patient became unresponsive. He was promptly ventilated with an ambulatory bag, which increased his oxygen saturation to 100%. Subsequently, he was successfully intubated using video laryngoscopy, and anesthesia was induced by administering propofol (30 mg) without a paralytic agent. Sedation was maintained with a propofol infusion (20 mcg/kg/min). In addition, white blood cells, red blood cells, and hemoglobin values were 14.06 × 103/µL, 3.91 × 106/µL, and 11.1 g/dl, respectively. A chest X-ray shows slight engorgement of the pulmonary vasculature and the endotracheal tube tip located in the lower trachea (Figure 1). Arterial blood gases (ABGs) taken at that time revealed a pH level of 7.02 and PaCO_2_ of 92 mmHg, indicating respiratory acidosis and CO_2_ narcosis. After optimizing his ventilatory status, the patient was successfully extubated in the PACU prior to being transferred to the neuro-intensive care unit (NSICU). Upon leaving the PACU, the patient was maintained with a venturi mask set at 50% O_2_, ensuring that saturation levels remained above 95%. In the NSICU, caution was advised regarding the use of opioids and sedatives due to the patient's history of delayed emergence, somnolence, and acute respiratory failure. The services of an endocrinologist were enlisted to monitor hormone levels. Subsequently, ABGs showed persistently elevated PaCO_2_ (55-60 mmHg) with no identifiable cause. In addition, a chest X-ray showed improvement in the status of the pulmonary vasculature and clearest lung fields three days after the procedure (Figure 2). He was discharged on postoperative day 5 after he had maintained optimal hormonal levels.

## Discussion

The prevalence of patients with OSA is increasing due to growing rates of obesity and longevity. Fundamental mechanisms in OSA pathogenesis include upper airway neuromuscular impairment, changes in respiratory arousal threshold, and ventilatory control instability. Anesthesia, analgesia, and surgery, individually or in combination, may disrupt ventilatory response and sleep architecture in patients with OSA. Indeed, there is an increase in AHI on the first night after surgery, with a peak on the third night [9].

Although the degree of severity of OSA may be a predictor of potential adverse events, in the current case, the patient was previously diagnosed with the condition and a polysomnography was not part of the preoperative workup. Nevertheless, during the perioperative period, all indications for the treatment of severe OSA were followed. Among patients with known OSA, approximately 10-20% also have a prevalence of OHS [10]. This subset of patients is more likely to experience respiratory failure, cardiac failure, and an unexpected stay in the intensive care unit [1]. Recognition of OHS helps avoid perioperative challenges that may include unnecessary oxygen supplements, OIVI, and worsening hypercapnia [11]. It also helps to incorporate documented approaches such as early positive pressure therapy that reduces mortality and postoperative complications [12]. Even though aspiration could occur in patients with these comorbidities, chest X-rays showed no evidence of aspiration in this case.

Adopting a pain management strategy that minimizes or spares the use of opioids is essential in mitigating the risk of OIVI. Patients with OSA and OHS have reduced central respiratory drive and are vulnerable to upper airway blockage, which places them at increased risk for OIVI [5]. Adhering to the ERAS (Enhanced Recovery After Surgery) Society recommendations regarding the utilization of various pain relievers in combination with regional analgesic techniques is of utmost importance in patients with OSA and OHS [13]. Non-opioid analgesics like IV lidocaine infusion, acetaminophen, and non-steroidal anti-inflammatory drugs may prove beneficial for pain management. If opioids are considered for analgesia, short-acting opioids or atypical opioids (weak mu receptor agonist), are preferred over longer-acting opioids. However, continuous monitoring of pulse oximetry and pain-sedation mismatches is required.

Low doses of ketamine and dexmedetomidine are also ideal agents for pain management in this subset of patients, as both drugs can preserve airway reflexes, reduce opioid consumption, and improve pain control [13]. When compared to acetaminophen alone, dexmedetomidine contains a higher degree of anti-nociceptive effects. Caution should be exercised when utilizing dexmedetomidine; however, several studies have described episodes of apnea, airway obstruction, and impaired hypoxic control of breathing secondary to its sedative properties [14,15].

Recently, there has been an increase in the use of gabapentinoids for analgesic purposes in the perioperative period. The use of these drugs may prove useful during the perioperative period, provided that the necessary precautions are taken, as previous studies have reported an association between gabapentinoids use and an increase in AHI [16]. Moreover, a recent cohort study described an association between the concomitant use of gabapentinoids with opioids and an increased risk of opioid overdose in patients undergoing major surgery. However, the absolute risk of adverse events is low [17]. In our patient, analgesic management initially consisted of opioid-sparing medications. Owing to inadequate analgesia, however, a low dose of morphine (0.035 mg/kg) was administered for pain management, causing respiratory failure type II.

## Conclusions

This case highlights the importance of developing a perioperative opioid-sparing analgesic plan in a patient with OSA and OHS, who was at risk of developing respiratory complications. Although safety cannot be established with a single case, an individualized approach, together with an opioid-sparing multimodal analgesic plan, may be crucial to ensure safety in patients with these comorbidities. In addition, patients with OSA and comorbid conditions, such as OHS, should be carefully pre-evaluated and optimized to prevent perioperative complications. Performing a respiratory assessment in the PACU can also aid in identifying patients who require a higher level of care with continuous pulse oximetry monitoring.
